# Tibial Plateau Fracture With Use of Tibia Strut and Bone Filler in a 37-Year-Old Male: A Case Report

**DOI:** 10.7759/cureus.52913

**Published:** 2024-01-25

**Authors:** Wayne Ngo, Germain Craddock, Alex Frangenberg, Amber Park, Niladri Basu

**Affiliations:** 1 Medicine, Texas College of Osteopathic Medicine, University of North Texas Health Science Center, Fort Worth, USA; 2 Medicine, Lincoln Memorial University DeBusk College of Osteopathic Medicine, Knoxville, USA; 3 Orthopedics, Dallas Orthopaedic Trauma Institute, Dallas, USA

**Keywords:** schatzker tibial plateau classification, tibial osteotomy, tibial plateau fracture, comminuted bicondylar tibial plateau fracture, orthopedics

## Abstract

Tibial plateau fractures (TPFs) are orthopedic challenges with multiple injury modalities and clinical presentations. TPFs are often classified using the Schatzker classification system, which can dictate management. In our case, a 37-year-old male presented at an orthopedic specialty hospital with right knee pain after a fall from a truck ramp. X-rays and CT imaging demonstrated a comminuted bicondylar TPF in the emergency room with metaphyseal dissociation. The patient was placed in a knee immobilizer, made non-weight bearing, and scheduled for outpatient follow-up with a local orthopedic surgeon. The patient was lost to follow-up and referred to our clinic six months after the fall with the chief complaint of inability to ambulate with severe pain and instability in the knee. X-rays demonstrated a malunion of the bicondylar tibial plateau with fracture deformities of the medial femoral condyle and lateral tibial plateau. The patient's deformity was corrected with a medial opening wedge proximal tibial osteotomy with a fibula strut allograft and filled with beta-tricalcium bone filler.

At the first month follow-up, the patient's pain was well controlled, fragments and the knee were appropriately aligned, and no significant soft tissue or joint effusion was appreciated on imaging. After failing nonoperative treatment, this patient with comminuted bicondylar TPF has received definitive treatment with open reduction and internal fixation. Higher rates of unacceptable results from nonoperative treatment are in line with the Schatzker series, in which operative treatment resulted in more acceptable outcomes. Because the fracture in this patient is consistent with a Schatzker VI classification with intra-articular depression, the patient should have initially been treated with an external fixator and not been sent home in a knee immobilizer. This led to a malunion that necessitated corrective surgery. Therefore, correctly classifying fracture severity is important for selecting the best treatment course and minimizing complications.

## Introduction

Tibial plateau fractures (TPFs) are orthopedic challenges with multiple injury modalities and clinical presentations. Because these fractures can have varying morphologies, a classification system is often necessary for the determination of appropriate treatment [1]. The most common way to classify TPFs is the Schatzker classification system which is based on a two-dimensional representation of the fractures and is organized by the patient's age, bone quality, fracture morphology, and the energy of the trauma [2-6].

Schatzker type I, II, and III fractures are often seen in low-energy trauma, involve the lateral tibial plateau, and are associated with mild to moderate soft tissue injury. In terms of fracture morphology, type I is described as a split wedge fracture of the lateral tibial plateau that is commonly seen in younger individuals who may have a higher proportion of cancellous bone [4]. Type I fractures can be managed with open reduction internal fixation (ORIF) using lag screws or buttress plates [3,4]. Type II is described as a split wedge fracture with depression, and management typically involves buttress plating. Type III is a pure depression fracture often seen in low-energy trauma involving individuals with decreased bone density. Surgical treatment of type III requires bone tamping, bone graft, and raft screws [4]. 

In contrast, types IV, V, and VI are associated with high-energy trauma and significant soft tissue compromise. These types of fractures often require spanning external fixation as an initial treatment to stabilize fracture fragments [2-4]. Type IV is an isolated fracture of the medial tibial condyle caused by varus shearing and can present with varying morphology. It is commonly associated with avulsion of the tibial spine and ligamentous injury and requires buttress plating for treatment. Type V is a bicondylar fracture of both the medial and lateral tibial condyles and also requires buttressing plating. Finally, type VI is similar to type V but is distinguished by disruption of the tibial rim and metaphyseal-diaphyseal dissociation [4]. For management, the tibial rim requires stabilization with buttress plating, while the shaft requires a bridging plate.

In general, operative management remains the gold standard for TPFs, as the torsion, rotation, and general stress the tibial plateau experiences leave little room for weight bearing in nonoperative patients before non-union or other complications may occur [7]. Usually, surgical management is indicated in displacement over 10 degrees of either varus or valgus, intra-articular displacement over or equal to 2 mm, or metaphyseal-diaphyseal translation over 1 cm [8,9]. However, existing literature on surgical management strategies of TPFs is limited in terms of comparison of nonoperative versus operative management. The ability to recognize factors and indications for either operative or nonoperative can help to determine the best treatment course for a patient with minimal complications. This case report highlights a scenario in which an incorrect treatment plan along with a loss to follow-up for a TPF led to a malunion requiring more extensive surgical correction. 

This article was previously presented as a meeting abstract at the 2023 University of North Texas Health Science Center Research Appreciation Day on March 27-31, 2023.

## Case presentation

**Figure 1:**
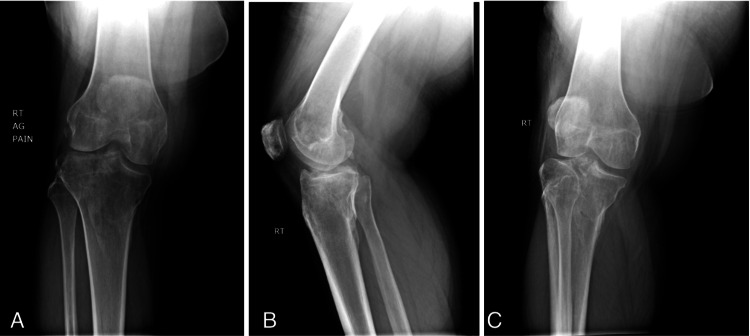
An anteroposterior (A), lateral (B), and oblique (C) X-ray of the right knee shows a complex bicondylar tibial plateau fracture with significant varus deformity after nonoperative management.

A 37-year-old male presented at an orthopedic specialty hospital with right knee pain after a fall from a truck ramp. The patient heard a pop and had severe, sharp pain in his right knee. In the emergency room, X-rays and CT imaging demonstrated a comminuted bicondylar TPF with metaphyseal dissociation. The medial tibial plateau was depressed by 5 mm. The patient was placed in a knee immobilizer, made non-weight bearing, and scheduled for outpatient follow-up with a local orthopedic surgeon. The patient was lost to follow-up and referred to our clinic six months after the fall. His chief complaint was an inability to ambulate with severe pain and instability in the knee. X-rays demonstrated a malunion of the bicondylar tibial plateau and depressed medial plateau with possible increased condylar width and varus alignment (Figure 1A-1C). CT scans showed healed fractures, and an MRI was ordered to rule out osteochondral defects and meniscus tears. The patient was indicated for corrective osteotomy.

**Figure 2:**
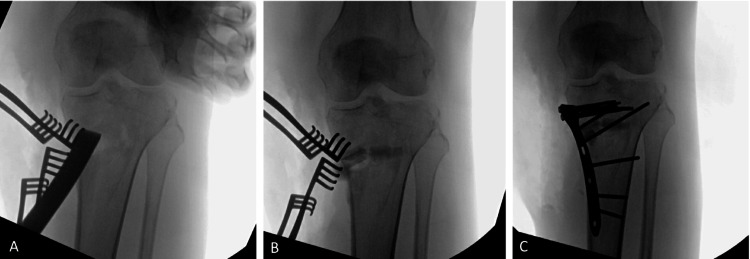
Intraoperative X-ray series showing a medial opening wedge proximal tibial osteotomy. Image A shows the elevation of the proximal tibia. Image B demonstrates the placement of the fibula strut. Image C shows the final position of the plate and screws.

The surgical technique was a medial opening wedge proximal tibial osteotomy. The patient was placed in a prone position, and an incision was made centered over the medial aspect of the gastrocnemius. The medial soft tissue sleeve was elevated off the proximal tibia in a subperiosteal fashion while maintaining the medial collateral ligament (MCL) insertions (Figure 2A). Next, a sagittal saw was used to make a medial tibial osteotomy. The medial proximal tibia was elevated to create an opening wedge osteotomy to correct varus deformity. Afterwards, a fibula strut allograft with a plate/screw construct was placed (Figure 2B). Beta-tricalcium bone filler was used to fill the void (Figure 2C). Postoperatively, the patient was made non-weight bearing for three months. Range of the motion was encouraged as tolerated.

**Figure 3:**
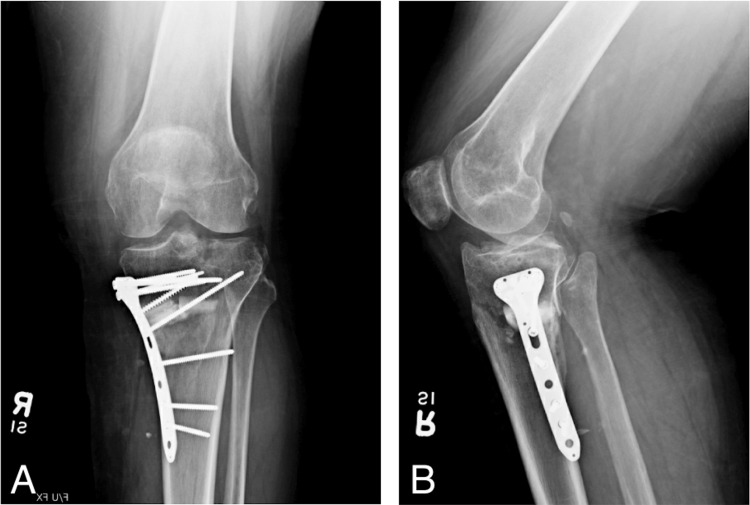
One-month follow-up X-rays of the right knee demonstrating intact hardware with appropriate alignment of the knee and fracture fragments.

At the first month follow-up, the patient's pain was well controlled, and range of motion exercises were done regularly. No paresthesia, numbness, or wound dehiscence was noted. Repeat X-rays demonstrated intact hardware with evidence of healing fracture lines compared to immediate postoperative images (Figure 3A, 3B). Fragments and the knee were appropriately aligned. No significant soft tissue or joint effusion was appreciated on imaging.

## Discussion

After failing nonoperative treatment, this patient with comminuted bicondylar TPF has received definitive treatment with ORIF. Higher rates of unacceptable results from nonoperative treatment align with the Schatzker series, in which operative treatment resulted in more acceptable outcomes [5,10]. Because the fracture in this patient is consistent with a Schatzker VI classification with intra-articular depression, the patient should have initially been treated with an external fixator and not been sent home in a knee immobilizer (Table 1). This led to a malunion that necessitated corrective surgery [8].

**Table 1:** The Schatzker classification system and surgical management.

Types	Anatomical classification	Treatment
Type I	Split wedge fracture of the lateral tibial plateau	Lag screw or buttress plate
Type II	Split wedge fracture with depression of the lateral tibial plateau	Buttress plate
Type III	Pure depression of the lateral tibial plateau	Bone tamping, bone graft, and raft screws
Type IV	Medial tibial condyle fracture	Buttress plate
Type V	Bicondylar fracture	Buttress plate
Type VI	Bicondylar fracture with tibial rim and shaft dissociation	Buttress plate and bridging plate

Although the standard treatment for a Schatzker type VI fracture involves buttress plating, a wedge osteotomy was required in this case to correct the chronic fracture deformity from the prior treatment plan. The challenge in this case was managing the subchondral void acquired after the elevation of the depressed medial tibial plateau. Various materials can fill this void, including autografts from the iliac crest, allografts, xenografts, and bone substitutes such as polymers and synthetics [11]. Autologous bone graft is considered the gold standard because it easily meets the standards of structural similarity to bone, biocompatibility, and osteoconductivity. However, pain and other complications at the donor site limit its use and invite alternatives [11]. This case study demonstrates that allograft could be an appropriate option for TPFs requiring wedge osteotomy.

There have been novel techniques for TPF treatment such as a novel fenestrated screw system that allows injection of bone graft substitute (e.g., calcium phosphate) to fill the subchondral void. Telis et al. have demonstrated with a 34-patient cohort that the rate of articular subsidence was similar to conventional methods, and they concluded it was a viable option for TPF treatment. [12]. Another alternative is arthroscopy-assisted reduction and internal fixation (ARIF) which is demonstrated as a reliable and effective method for TPF treatment, especially with concomitant anterior cruciate ligament and menisci injuries by Chen et al. [13].

## Conclusions

Current management of TPF can include nonoperative and operative management. Patients should be thoroughly advised on the need for follow-up to avoid complications such as malunion. Discharging a patient without stabilizing the fracture can lead to catastrophic consequences such as those from this patient's initial treatment. Surgical treatment with ORIF remains the gold standard and can provide adequate healing and stability, as demonstrated in this case and the literature. There are standard choices in filling material for subchondral voids, but novel and promising solutions are on the horizon.
